# Research of shRNAmir inhibitory effects towards focal adhesion kinase expression in the treatment of gastric cancer

**DOI:** 10.3892/ol.2014.2725

**Published:** 2014-11-21

**Authors:** GUO-QIANG SU, FU-XING ZHANG, HE-HUI MAO, XIAN-WEI LIU, YONG-SHENG ZHENG, SI-YU ZHANG, JING-JUN SU

**Affiliations:** 1 Department of General Surgery, The First Affiliated Hospital, Xiamen University, Xiamen, Fujian 361003, P.R. China; 2 Department of Ultrasound Diagnosis, The First Affiliated Hospital, Xiamen University, Xiamen, Fujian 361003, P.R. China

**Keywords:** focal adhesion kinase, gastric cancer, RNAi, invasion, metastasis, nude mouse

## Abstract

Gastric cancer is the fourth most common type of malignant tumor, with a poor prognosis. Focal adhesion kinase (FAK) mediates the crosslink of intracellular signaling networks, playing a key role in cell migration and invasion. The aim of the present study was to investigate the effects of FAK interference on the proliferation ability, invasion and metastasis of gastric cancer cells. The FAK-RNAi lentiviral vector was infected into SGC7901 gastric cancer cells in order to observe the *in vivo* situations of tumor growth and metastasis before and after the FAK interference. The growth of SGC7901 gastric cancer cells in the interference group was significantly inhibited compared with that of the negative control (P<0.05) and the blank control groups (P<0.05), and the FAK expression significantly decreased (P<0.05). The *in vitro* invasion and metastasis experiments showed that the cell invasion and metastasis abilities of the interference group significantly decreased when compared with those of the negative control (P<0.05) and blank control groups (P<0.05). In the nude mouse subcutaneous tumor transplantation model, the mean ± standard deviation tumor weight of the interference group (1.474±0.9840 g) was lower than that of the negative control (3.134±0.3299 g) and blank control (2.68±0.12 g) groups (P<0.05). In the nude mice, the liver and peritoneal metastasis rates of the interference group were significantly lower than those of the negative control (P<0.05) and the blank control groups (P<0.05), and the FAK mRNA of the interference group significantly reduced (P<0.05). In conclusion, FAK interference could effectively suppress the proliferation, invasion and metastasis of transfected SGC7901 gastric cancer cells, and could inhibit the growth and distant metastasis of gastric cancer in nude mice.

## Introduction

Gastric cancer is the fourth most common malignant tumor (1) and has a poor prognosis; the 5-year survival rate is usually ≤20% (2). Invasion and metastasis are the main reasons for clinical treatment failure and patient mortality (3–5). In addition, the current clinical therapies do not obtain good outcomes. Therefore, novel therapies for the diagnosis and treatment of gastric cancer are required. It has been shown that gastric cancer metastasis or invasion of the adjacent tissues via penetration of the stomach wall is a complex process, which is associated with numerous factors and includes steps, such as tumor cell invasion, breaking through the cellular matrix, entering the blood, reaching distant organs, settling and proliferating (6). As an important intracellular signaling molecule, focal adhesion kinase (FAK) mediates the crosslink of the intracellular signaling networks, playing a key role in cell migration and invasion. Su *et al* (7) found that compared with non-cancerous tissues, FAK expression in gastric cancer tissues increased. In particular, FAK expression in poorly differentiated gastric cancer tissues was increased compared with that in well-differentiated cancer tissues; FAK expression in the tumors with lymph node metastasis was enhanced compared with that in those without lymph node metastasis; and the deeper the extent of tumor invasion, the stronger the FAK expression. Numerous studies have reported that the expression of FAK is increased in the invasive and metastatic tumors (8–15). The high expression of FAK is suggested to promote the migration and metastasis of tumor cells, therefore, interfering with the functions of FAK may provide new insights into gastric cancer therapy.

In the present study, RNA interference (RNAi) technology and novel lentiviral vector were used to prepare the recombinant FAK-shRNA lentivirus for infecting the human metastatic gastric cancer cells. *In vivo* and *in vitro* investigations were performed to observe the growth and metastasis of gastric tumors following the intervention of FAK functions, aiming to provide the basis for new gastric cancer therapies. This study was approved by the ethics committee of the First Affiliated Hospital of Xiamen University (Xiamen, China).

## Materials and methods

### Construction of FAK interference vector

The pLentilox3.7 plasmid was kindly provided by Professor Boan Li, Xiamen University (Xiamen, China). The primer design and vector construction used to intervene with FAK gene expression were as described previously (16). The positive plasmids identified by enzyme-cutting were sent to Shanghai Invitrogen Biotechnology Co., Ltd. (Shanghai, China) for the nucleotide sequencing.

### Lentiviral packaging and titer determination

According to reported methods (17), SGC7901 cells (Shanghai Institute of Biochemistry and Cell Biology, Shanghai, China) were cultured, and the cells with 80% density were used for transfection. A total of 5 ml of serum-free Dulbeccos’s modified Eagle’s medium (DMEM) was added to the cells, followed by the addition of the transfection reagent system (Shanghai Run-Biotech Co., Ltd., Shanghai, China) and incubation for 6 h. Serum-free DMEM was then replaced with DMEM with 2% fetal bovine serum (Shanghai Jiang Lai Biotechnology Co., Ltd., Shanghai, China), followed by incubation for 48 h. Next, the cell supernatant was collected, followed by centrifugation at 1,030 × g for 5 min at 4°C (3–18K; Sigma-Aldrich, St. Louis, MO, USA) and filtration. The supernatant was removed, and 1 ml of medium was added. The mixture was placed in a refrigerator at 4°C until the virus was dissolved completely. After sub-packaging, the virus liquid was placed in a refrigerator (Qingdao Haier Co., Ltd., Qingdao, China) at −80°C for use. The virus supernatant was diluted with serum-free DMEM. The diluted virus liquid was then mixed with the SGC7901 cells with 90–100% density, followed by incubation in 5% CO_2_ at 37°C. The cells were counted under a fluorescent microscope (DMS-853; Shenzhen Boyu Instrument Co., Ltd., Shenzhen, China). The virus titer was determiend using the following formula: Virus titer (IU/mL) = number of fluorescent cells/ml suspension × dilution multiple.

### Establishment of stably transfected gastric cancer cells SGC-7901

#### Recombinant lentivirus plasmid-transfected SGC-7901 cells

The cells in the logarithmic growth phase were inoculated into a 10 cm-dish and grown to ~70% confluence for the transfection. According to the different structures of the lentiviruses, the cells were divided into three groups: The interference experimental group was infected with FAK-shRNA lentivirus, the negative control group was infected with empty lentiviral vector and the blank control group received no treatment. The constructed lentiviruses were used to transfect the SGC-7901 cells (Cell Bank of Shanghai Institute of Cell Biology, Chinese Academy of Sciences, Shanghai, China) in the logarithmic growth phase according to the multiplicity of infection (MOI), 10 μl of 1,000X polybrene (Gibco Inc., Billings, MT, USA) was added and the medium was replaced after 24 h. Subsequently, RPMI-1640 medium (Gibco Inc.) was added to complete medium for another 48 h of incubation, prior to using a fluorescence microscope (BX51; Olympus Corporation, Tokyo, Japan) to observe the transfection efficiency. Western blotting was used to analyze the FAK expression.

### Screening of stable recombinant lentiviral plasmid-transfected SGC-7901 cells

SGC-7901 cells were counted and 1×10^5^ cells were plated in 10 wells of a 24-well plate, which were sequentially labeled as 1–10. The medium was replaced on the following day and, when the cells reached the logarithmic growth phase, different concentrations of neomycin G418 (Gibco Inc.) were added. The G418 concentration was 100 μg/ml in well no. 1 and increased to 1,000 μg/ml in well no. 10, arithmetically. The medium was replaced after 24 h and the cell growth was observed. The lowest concentration that could completely kill the cells on the 14th day was used as the screening concentration.

### In vitro experimental grouping

SGC7901 gastric cancer cells were divided into the interference group (IG; infected with FAK-shRNA lentivirus), the transfected negative control group (TN; infected with empty lentiviral vector) and the untreated group (UG).

### In vitro invasion and migration assay

After 48 h transfection, the cells were digested with trypsin (Beijing CellChip Biotechnology Co., Ltd., Beijing, China) and, following this, the culture medium was removed by centrifugation at 716 × g for 5 min. Subsequently, the cells were resuspended with serum-free medium for cell counting and the cell density was adjusted to 5×10^4^ cells/well. The Matrigel basement membrane matrix (BD Biosciences, Franklin Lakes, NJ, USA) was added to the Transwell chamber, followed by incubation at 37°C for 2 h. A total of 1 ml cell suspension was then added into a Transwell chamber and the cells were cultured at 37°C and 5% CO_2_ for 48 h. Following this, the Transwell chamber was removed, and a cotton swab was used to remove the cells on the Matrigel side of the Transwell chamber. Next, 0.1% crystal violet (Shanghai Zerun Biotechnology Co., Ltd., Shanghai, China) was used for staining for 1 h. The film of the Transwell chamber was rinsed with double distilled water, and removed for affixation to the back of the slide for cell counting under an inverted microscope (CKX31; Olympus Corporation, Tokyo, Japan). The procedures of the migration assay were the same as the invasion assay, with the exception of using the Matrigel basement membrane matrix.

### In vivo cell lines and experimental animals

SGC-7901 gastric cancer cells were purchased from the Cell Bank of Shanghai Institute of Cell Biology, Chinese Academy of Sciences. The SGC-7901 cells were divided into the same three groups as previously: IG, TN and UG. BALB/C female nude mice were purchased from Shanghai SLAC Laboratory Animal Co. Ltd. (Shanghai, China), were aged 4 to 6 weeks and weighed 16–19 g [license no. SCXK (Hu) 2007-0005]. The mice were fed under SPF conditions in the Cancer Research Center of Xiamen University for 1 week before the experiment.

### Establishment of in vivo animal model

#### Animal grouping and cell preparation

60 BALB/C/nu mice were randomly divided into six groups and labeled as Groups 1–6 (n=10 for each group). Groups 1–3 were the subcutaneous tumor transplantation models and Groups 4–6 were the orthotopic tumor transplantation models. IG, TN and UG cells were trypsin-digested into a single cell suspension, washed three times with RPMI 1640 medium and then counted under microscope (CKX41; Olympus Corporation).

#### Establishment of the subcutaneous tumor transplantation model

The cell density was adjusted to 1×10^7^, then 0.2 ml IG, TN and UG cells were inoculated subcutaneously into the armpits of mice in Groups 1–3, respectively. The injection method involved conventional disinfection of the skin with alcohol, and then the needle was inserted in the chest ~2 cm from the right armpit, reaching the right armpit along the subcutaneous tissue. The cell suspension was slowly injected and, on removal of the needle, an alcohol swab was compressed on the area to avoid leakage of the injected tumor cells.

#### Establishment of the orthotopic tumor transplantation model

Mice in Groups 4–6 were initially fasted for 12 h. IG cells were inoculated first: Following anesthesia with ether, the abdominal cavity was opened to reveal the stomach and 0.1 ml cell suspension was inoculated under the mucous membrane of the greater curvature of the gastric antrum. The bulge under the serous membrane of the stomach was used to confirm successful vaccination. A sterile cotton ball was pressed onto the injection site for 1 min following the removal of the needle, to ensure that the cells did not leak out into the abdominal cavity. Suture of the peritoneum, muscle and skin was performed layer by layer. Postoperative disinfection with 75% alcohol and iodine was conducted and then the mice were put back into the cages. The same method was used to inoculate the TN and UG cells.

#### Rearing observation

Following inoculation, both the subcutaneous and orthotropic mice were bred in a SPF animal room, and regular observation of activity and eating habits were performed. The body weights and armpit tumor volumes in the nude mice were measured and evaluated. The long diameter (a) and short diameter (b) of tumors were measured with a vernier caliper for the calculation of the approximate tumor volume (V) (17), according to the formula V (mm^3^) = a × b^2^/2. Through observation, all mice were alive and exhibited a tumor 1 week after the inoculation, with the tumor formation rate as 100%. During the rearing process, there was no mortality in Groups 1–3, while two mice in each of Groups 4–6 died on the 10th day after inoculation. All mice were sacrificed (by decapitation) after 4 weeks of feeding.

### Tumor resection and tissue analysis

The tumors were resected and their size and weights were measured. The Carestream imaging system (Eastman Kodak Company, Rochester, NY, USA) was used for fluorescence detection of tumor *in vitro*. Simultaneously, laparotomy and thoracotomy were performed to obtain the liver and lungs of the mice. The thoracic cavity was dissected to investigate whether tumor metastasis had occurred. The tumor tissue paraffin sections were prepared and dewaxed followed by antigen retrieval. The primary rabbit anti-human FAK monoclonal antibody (Wuhan Boster Biological Technology, Ltd., Wuhan, China) was added, followed by incubation at room temperature for 90 min. After two washes with PBS, the horseradish peroxidase-conjugated goat anti-mouse IgG secondary antibody (Fuzhou Maixin Biotechnology Development Co., Ltd., Fuzhou, China) was added, followed by incubation at room temperature for 15 min and two washes with PBS. After coloration, counterstaining and mounting, the sections were observed using the Q550CW image acquisition and analysis system (Leica, Mannheim, Germany). The primary antibody was replaced with PBS, to serve as a negative control and the known positive tissue sample was used as positive control. Each section was divided into five fields of vision (×200), with 100 counted cells in each field using the Q550CW image acquisition and analysis system (Leica). The percentage of positive cells was scored as follows: 0, 0–5%; 1, 6–25%; 2, 26–50%; 3, 51–75%; and 4, >75%. The staining intensity was scored as follows: 0, no staining; 1, weak staining intensity; 2, moderate staining intensity; and 3, strong staining intensity. The percentage score and staining intensity scores were then added to obtain a total staining score, which was scored as follows: <2, negative (−); 2–3, weakly positive (+); 4–5, positive (++); and 6–7, strong positive (+++). Quantitative polymerase chain reaction (qPCR) was conducted according to a previously reported method (18). The total RNA was extracted from the tumor tissue. The PCR-reaction mixture containing 1 μg total RNA was added to a 200 μl PCR tube. The primer sequences for FAK (125 bp) were as follows: Forward, 5′-ACATTATTGGCCACTGTGGATGAG-3′ and reverse, anti-sense primer: 5′-GGCCAGTTTCATCTT GTTGATGAG-3′ for FAK (125 bp); and forward, 5′-GATGCAGAAGGAGATCACTG-3′ and reverse, 5′-GGGTGTAACGCAACTAAGTC-3′ for the reference, β-actin (222 bp). All primers were synthesized by Shanghai Invitrogen Biotechnology Co., Ltd. (Shanghai, China). The PCR steps were as follows: initial denaturation for 3 min at 94°C, denaturation for 30 sec at 94°C, annealing for 30 sec at 59°C and elongation for 1 min at 72°C for 35 cycles, followed by continued elongation at 72°C for 7 min.

### Statistical processing

SPSS 13.0 software (SPSS, Inc., Chicago, IL, USA) was used for data processing. The tumor cell proliferation and tumor weight in the different groups were compared using single-factor analysis of variance, while FAK expression and tumor metastasis were compared using χ^2^ and Fisher’s exact tests. P<0.05 was considered to indicate a statistically significant difference.

## Results

### Construction, identification and viral packaging of vector

**Figure 1:**
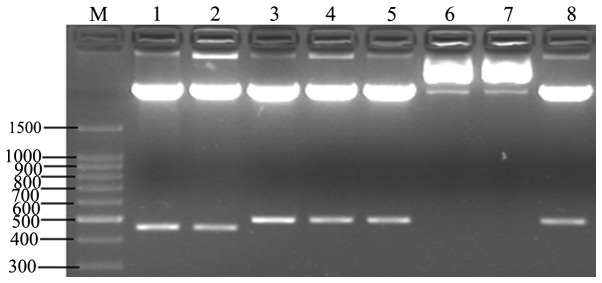
Enzyme digestion identification of pLentilox3.7 FAK positive recombinant plasmid and the negative control recombinant plasmid. M, 100-bp ladder DNA marker; 1, pLentilox3.7 empty plasmid; 2–4; negative control recombinant plasmid; 5–8, pLentilox3.7 FAK recombinant plasmid. FAK, focal adhesion kinase.

The amplified plasmid was identified by *Xba*I and *Not*I digestion electrophoresis. As shown in Fig. 1, the FAK-RNAi recombinant plasmid obtains a 504-bp product by double digestion (insert fragment size, 55 bp); while the pLentilox3.7 empty vector, which was inserted, obtains a 449-bp product after the double digestion (lane 1). Lane 2 revealed that the target gene was not successfully inserted, while lanes 6 and 7 showed that the enzyme digestion of recombinant plasmid was not successful. The results revealed that the product identification results of lanes 3, 4, 5 and 8 were the same as the expectations, i.e. plasmids from the positive colonies. Lanes 3 and 4 indicated the successful construction of the negative control plasmids, while lanes 5 and 8 exhibited the positive colony plasmids. Certain plasmids from the positive colonies were sequenced by Shanghai Invitrogen Biotechnology Co., Ltd., and the results showed that they contained the correct target gene sequence (Fig. 1). The results showed that FAK shRNA and control shRNA were constructed in the pLentilox3.7 vector, and the recombinant was constructed successfully. After packaging with 293T cells, the titer of the recombinant virus group was 4×10^7^ pfu/ml.

### Screening concentrations of G418

After 14 days, cells in well nos. 7, 8, 9 and 10, i.e. wells with a G418 concentration of 700, 800, 900 and 1,000 μg/ml, respectively, were all dead. Additionally, only trace cells survived in the 600 μg/ml G418 group.

### Establishment of a stable FAk-silencing SGC-7901 cell line

**Figure 2:**
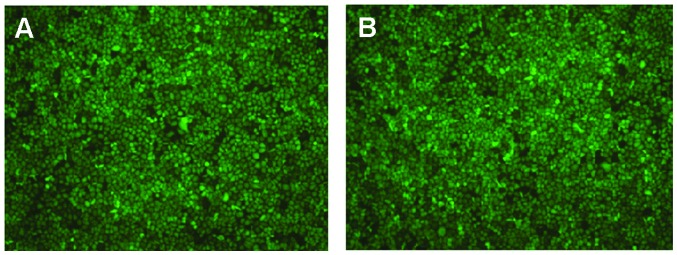
Fluorescence images of the screening of the recombinant lentivirus infection 14 days following transfection (magnification, ×200) in (A) interference group and (B) transfected negative control group cells.

During the cultivation of the transfected recombinant lentivirus SGC-7901 cells in each group, the G418 concentration was maintained at 600 μg/ml for consecutive 14 days, the transfected cells grew well and the fluorescence expression increased (Fig. 2).

### Western blot analysis of FAK expression

**Figure 3:**
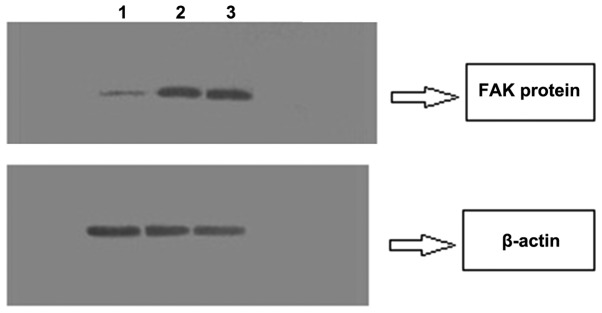
Western blot analysis of FAK and β-actin protein expression in each group. FAK, focal adhesion kinase; 1, interference group; 2, transfected negative control group; 3, untreated group.

Compared with the TN and NG SGC-7901 cells, the FAK protein expression in the IG SGC-7901 cells significantly reduced, suggesting that the transfected recombinant lentivirus plasmid could effectively silence the FAK gene (Fig. 3).

### Effect of different MOIs on SGC-7901 cells

Following the infection with lentivirus, with increasing MOIs, the proportion of the infected cells also increased. When the MOI was 20, ~60% of cells were infected; when the MOI was 30, ~90% of cells were infected; and when the MOI was 40, a large number of cells began to die. The cell transfection experiments were therefore performed with an MOI of 30.

The optimal MOI of 30 was used to infect cells of each group, and it was found that compared with the TN and UG groups, the cell proliferation ability in IG cells (3.75±0.01) was significantly inhibited compared with that in TN (8.29±0.32) and NG (8.16±0.29) cells, respectively (P<0.05), and, over time, the difference gradually increased.

**Figure 4:**
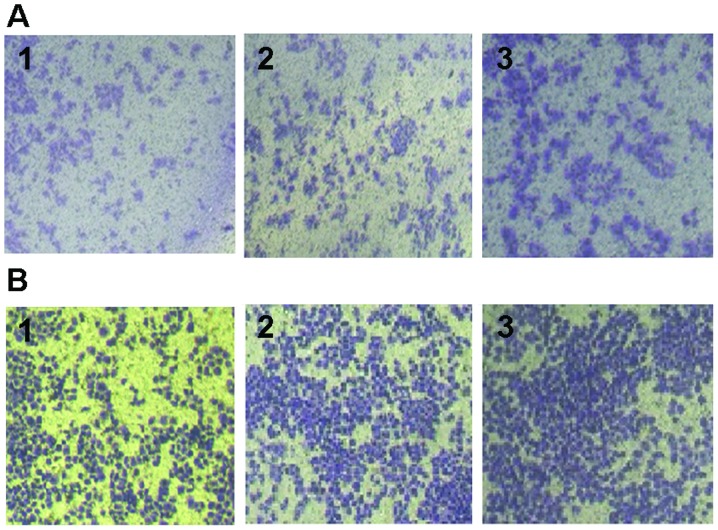
Invasiveness and metastasis of each group. (A) Cellular invasiveness determination and (B) cellular metastasis test. 1, interference group; 2, transfected negative control group; 3, untreated group (magnification, ×100).

In the Transwell chamber invasion assay, the number of membrane-penetrating IG cells (59.3±4.1) was significantly lower than that of TN (105.1±3.7) (P<0.05) and UG (103.8±8.3) (P<0.05) cells. There was no significant difference between that of TN and UG (P>0.05) cells, indicating that the cell invasiveness in IG cells was significantly inhibited (Fig. 4).

### In vivo tumor formation rate of each subcutaneous tumor transplantation group

Observation of the subcutaneous and orthotopic tumor transplantation groups revealed that on the seventh day of tumor cell inoculation, tumors of 2 mm in diameter were visible in each group, and the tumor formation rate was 100%.

### Comparison of tumor formation in vivo in each subcutaneous tumor transplantation group

The mice were sacrificed 4 weeks following inoculation and the mean±standard deviation (SD) average tumor weight in the IG, TN and UG inoculation groups was 1.474±0.9840, 3.134±0.3299 and 2.687±0.3827 g, respectively. The mean (±SD) tumor weight in the IG inoculation group was significantly smaller than that of the remaining two groups (F=18.09, P=0.0017).

**Figure 5:**
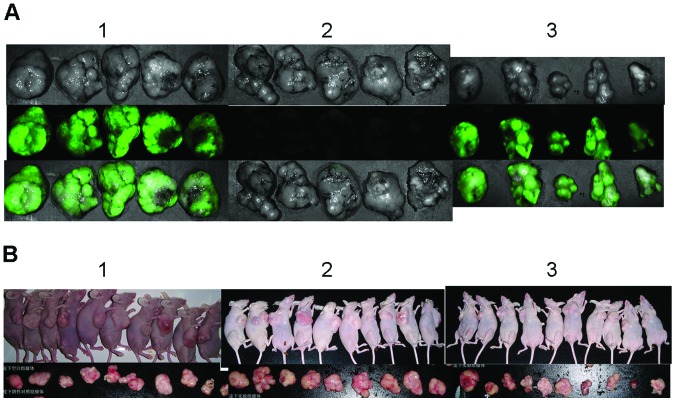
(A) Tumor fluorescence and weight of each subcutaneous tumor transplantation group. (B) Groups 1–3: Mice subcutaneously injected with interference group, transfected negative control group and untreated group cells, respectively.

Fluorescence detection of the tumor *in vitro* revealed that there was no strong fluorescence expression in the IG and TN inoculation groups, while no fluorescence expression was identified in mice inoculated with UG cells. This indicated that the virus-packaged plasmid, labeled with green fluorescent protein, infected the SGC-7901 gastric cancer cells, the cells repeatedly proliferated in nude mice and the target gene expression remained strong (Fig. 5).

qPCR revealed statistically significant difference in FAK mRNA expression levels between the IG and TN or UG inoculation groups (P<0.05; Table I).

### Comparison of stained tumor sections in vivo in each subcutaneous tumor transplantation group

**Figure 6:**
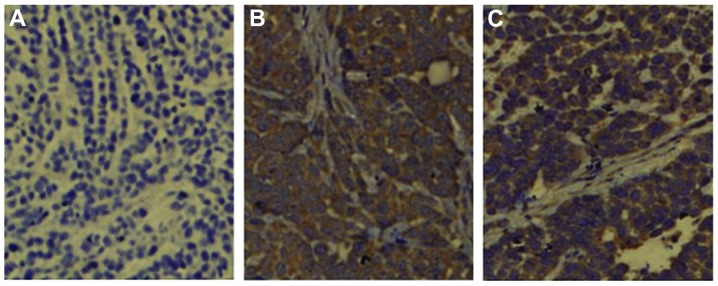
Immunohistochemical staining results of each subcutaneous tumor transplantation group (magnification, ×200). (A) Interference group, (B) transfected negative control group and (C) untreated group.

Positive expression of FAK was identified in the TN and UG groups, while almost no expression of FAK was observed in the IG group (Fig. 6).

### Comparison of metastasis in vivo in each orthotopic tumor transplantation group

Eight nude mice survived in each group: One mouse exhibited hepatic metastasis and no mice exhibited peritoneal metastasis in the IG inoculation group; six mice demonstrated hepatic metastasis, while four had peritoneal metastasis in the TN inoculation group; five mice had hepatic metastasis and four showed peritoneal metastasis in the UG inoculation group. The difference was statistically significant between Group 1 and Groups 2 and 3 (P<0.05). The FAK mRNA levels in Group 4 were significantly lower than those in Groups 5 and 6.

**Figure 7:**
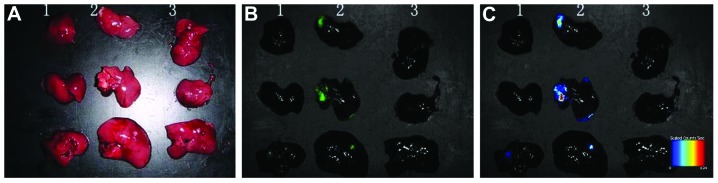
Fluorescence expression of hepatic metastasis lesions of each orthotopic tumor transplantation group. (A) Hepatic metastasis observed with the naked eye, and (B) fluorescence imaging and (C) fluorescence density expression photographs of hepatic metastasis lesions. 1, interference group; 2, transfected negative control group; 3, untreated group.

**Figure 8:**
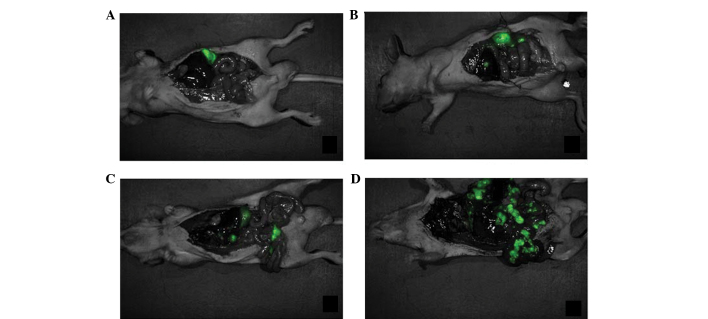
Fluorescence expression of orthotopic and metastatic lesions of each orthotopic tumor transplantation group. (A) Fluorescence could only be seen in the orthotopic tumor, while no fluorescence was observed in the peritoneal metastasis of mice inoculated with interference group cells. (B, C and D) Fluorescence in the peritoneal metastasis of mice inoculated with transfected negative control group cells. Data for the untreated group data is not shown.

**Figure 9:**
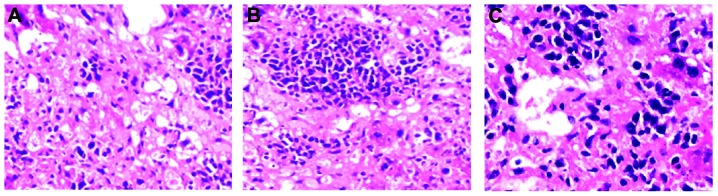
Hematoxylin and eosin staining results of the hepatic metastases of gastric tumors in each orthotopic tumor transplant group inoculated with (A) interference group cells (magnification, ×100), (B) transfected negative control cells (magnification, ×100) and (C) untreated cells (magnification, ×200).

3D fluorescence imaging could better display the fluorescence in the liver and peritoneal metastases. A significant difference was identified in the levels of liver and peritoneal metastasis of the mice inoculated with IG and TN or UG cells (Figs. 7 and 8). The immunohistochemical staining of metastatic lesions revealed liver metastasis of gastric cancer in mice inoculated with TN and UG cells (Fig. 9).

## Discussion

The expression of FAK increases in the early stage of tumorigenesis, resulting in the potential of invasion and metastasis of tumor cells. When the expression of FAK is blocked, the apoptosis of tumor cells is then induced (18). Therefore, the present study aimed to investigate the changes in the biological behavior of gastric cancer cells in which FAK function had been knocked down or out. Firstly, high-efficiency transfection technology and hairpin RNA was used to build the FAK interference vector (shRNA lentivirus). The study found that FAK-shRNA lentivirus could stably infect the SGC790 gastric cancer cells, and then significantly inhibit the expression of FAK following the infection. Then, through Transwell chamber migration and invasion assays, it was found that the membrane-penetrating IG cells significantly decreased compared with that of TN and UG cells, while there was no significant difference between the UG and TN cells, indicating that the inhibition of FAK expression could significantly decrease the invasion and migration of SGC7901 cells. The results are similar to those in a study by Ren *et al* (19), where siRNA was used to inhibit the expression of myofibrillogen-esis regulator 1 in human laryngeal carcinoma Hep-2 cells, thereby significantly reducing FAK phosphorylation at Tyr-925, and significantly inhibiting the invasion and metastasis of the Hep-2 cells. Additionally, Tan *et al* (18) found that the increased invasiveness of human chondrosarcoma cells by Cyr61 was likely through the signaling pathway that was dependent on ανβ integrin, FAK, ERK and AP-1. Furthermore, Hauck (21) found that the inhibition of FAK activity or blocking the expression of FAK could inhibit the invasion of tumor cells. Additional studies have confirmed that the inhibition of FAK expression could effectively reduce the adhesion and invasion of tumor cells (22,23), which is consistent with the results of the present study, suggesting that *in vitro* inhibition of FAK expression inhibits the invasion and migration of tumor cells.

To investigate the effects of FAK interference *in vivo*, a stably transfected gastric cancer cell model was established in this experiment. The monoclonal cells with a good interference effect and vigorous growth were selected and largely proliferated *in vitro* to be used as the *in vivo* interference group of the present study. Simultaneously, a negative control group (empty plasmid-transfected group) and a blank control group were also established, and cells of all three groups were transplanted into nude mice, respectively. A subcutaneous tumor transplantation model and an orthotopic tumor transplantation model were also established. The results showed that in each subcutaneous group, there were significant differences in the tumor volume and weight between mice inoculated with IG and TN/UG cells; while there was no significant difference in the tumor volume and weight between mice inoculated with TN and UG cells.

Furthermore, qPCR detection revealed that there was a significant difference in the FAK mRNA levels between the IG and TN/UG cell-inoculated mice, while there was no statistical significance between the latter two groups. Immunohistochemical staining showed that the expression of FAK in mice inoculated with IG cells was significantly lower than that of mice inoculated with TN and UG cells, which was consistent with the *in vitro* western blot results. Analysis of the results suggested that the shRNA in IG cells could effectively degrade FAK mRNA, thereby reducing or inhibiting the expression of FAK. This study showed that, in the subcutaneous tumor transplantation group of gastric cancer nude mice, the tumor growth was successfully inhibited, and FAK gene transcription and protein expression in tumor tissues were reduced. In the treatment of melanoma, Li et al(22) directly injected siRNA to intervene the intratumoral FAK plasmids, and it was found that when the FAK expression reduced, the average tumor weight in the mouse tumor model also decreased.

In the orthotopic tumor transplantation group, the gross anatomy of the nude mice was combined with an *in vitro* fluorescence imaging system to locate the metastatic lesions. The positioning was accurate and therefore a more accurate understanding of the tumor metastasis situation in nude mice was achieved. It was identified that there were significant differences in the levels of liver metastasis among IG, TN and UG cell-inoculated mice. There were significant differences in the levels of peritoneal metastasis between IG and TN/UG cell-inoculated mice, while there was no significant difference in the levels of peritoneal metastasis between TN and UG cell-inoculated mice. The H&E staining of the metastatic lesions showed that there was gastric cancer metastasis in the liver tissues. The orthotopic tumor transplantation model showed that when the FAK gene expression was silenced by RNAi technology, the metastasis of gastric cancer in nude mice was inhibited, which further confirmed that FAK plays an important role in the process of tumor metastasis, suggesting that orthotopic tumor transplantation may better present the biological characteristics of SGC-7901 cells in the event of metastasis. Further study with an increased sample size is required to investigate the significant difference in the peritoneal metastasis between IG and UG cell-inoculated mice that was identified in the present study.

Notably, during the establishment process of the subcutaneous tumor model, in order to avoid premature necrosis and ulceration of the tumors, careful observation was paid to the injected densities of SGC-7901 cells. It was found that when the inoculated concentration was 1×10^8^ cells/well, the grain-sized mass in the injection site was palpable 5 days after inoculation and the subcutaneous tumor would appear ulcerated or bleed 15 days following the inoculation. Additionally, the ulceration would form a scab if feeding was continued, the longitudinal growth rate would be lower than previously, and the tumor may grow around the vaccination site. By contrast, when the inoculated concentration of SGC-7901 was 1×10^7^ cells/well, a tumor was palpable in the injection site 7 days after inoculation. After 21 day, the skin covering the surface of the tumor was rosy and smooth, without rupture and bleeding. However, when the inoculated concentration was 1×10^6^ cells/well, tumor formation occurred 15 days after inoculation and the growth was slow. Therefore, the appropriate inoculation concentration was determined to be 1×10^7^ cells/well. These observations were similar to those identified previously (25,26), which provided a valuable insight into the establishment of animal gastric cancer models.

In summary, the present study successfully constructed a pLentilox3.7 FAK lentiviral vector and, through establishing the stably transfected cell lines, the *in vivo* and *in vitro* studies confirmed that the expression of FAK in SGC-7901 gastric cancer cells was reduced and the FAK-based signal transduction pathway was blocked, which could effectively inhibit the growth and metastasis of cancer cells. Therefore, the current study has provided new insights into clinical gene therapy for gastric cancer.

## Figures and Tables

**Table I:** Expression of FAK protein in tumor specimens of each group.

	FAK mRNA expression			
		
Group	+	++	+++	P-value
IG	7	2	1	0.034
TN	2	4	4	
UG	1	4	5	

FAK, focal adhesion kinase.
